# Supplementary material for: Prognostic Value of Mid-Regional Proadrenomedullin Sampled at Presentation and after 72 Hours in Septic Patients Presenting to the Emergency Department: An Observational Two-Center Study
Source: Biomedicines. 2022 Mar 19;10(3):719. doi: 10.3390/biomedicines10030719 (PMC8945269; doi:10.3390/biomedicines10030719)

Prognostic value of mid-regional proadrenomedullin sampled at presentation and after 72 hours, in septic patients presenting to the Emergency Department: an observational two-center study

SUPPLEMENTARY MATERIAL

Supplementary Table S1. Selected characteristics of patients with septic shock.

Supplementary Table S2. ROC curve analysis in patients with septic shock. P-values are calculated with respect to SOFA score.

Supplementary Table S3. ROC curve analysis in patients with a pulmonary infective focus. P-values are calculated with respect to SOFA score.

Supplementary Table S4. Logistic regression analysis.

Supplementary Table S5. Logistic regression analysis in patients with septic shock.

Supplementary Table S6. Logistic regression analysis in patients with a pulmonary infective focus.

Supplementary Figure S1. Flow diagram of the study.

Supplementary Figure S2. Dose-response plot for 28-day mortality predicted by MR-proADM$_{t0}$ (panel A) and MR-proADM$_{t72}$ (panel B) at varying levels of the APACHEII score.

Supplementary Table S1. Selected characteristics of patients with septic shock.

	**Total** (N=38)	**Survivors** (N=30)	**Non-Survivors** (N=8)	p-value
**Biomarkers at presentation**				
MR-pro-ADM (nmol/L)	4.40 (2.14-8.50)	4.35 (1.86-7.51)	7.83 (2.99-12.2)	0.25
PCT (ng/mL)	13.89 (5.06-77.02)<br>Missing 1	12.33 (5.01-77.02)	16.82 (4.93-75.85)	0.85
Lactate (mmol/L)	3.6 (2.0-5.5)<br>Missing 1	3.20 (1.90-5.00)	4.95 (2.55-7.55)	0.28
CRP (mg/L)	196 (63-281)	204 (84-284)	120 (23-266)	0.31
**Biomarkers at 72-hours from presentation**				
MR-pro-ADM (nmol/L)	2.94 (1.47-6.48)<br>Missing 2	2.31 (1.35-4.50)	7.24 (2.93-11.65)	0.03
PCT (ng/mL)	9.20 (2.68-21.70)<br>Missing 9	8.10 (1.97-20.78)	63.13 (26.26-100)	0.11
CRP (mg/L)	174 (109-248)<br>Missing 4	174 (111-284)	163 (66-284)	0.74
$proADM_{\%change}$	-19% (-55%, 5%)<br>Missing 2	-21% (-55%-4%)	4% (-14%,21%)	0.04
**Severity scores at presentation**				
SOFA score	6 (6-9)	6 (6-10)	6 (6-7)	0.29
APACHE II	24 (20-33)	23 (20-28)	36 (30-40)	0.008

Supplementary Table S2. ROC curve analysis in patients with septic shock. P-values are calculated with respect to SOFA score.

	**28-day Mortality**	
	AUC (95%CI)	P-value
SOFA	0.39 (0.21-0.57)	-
APACHEII	0.81 (0.59-1.0)	0.006
MR-pro-ADM$_{t0}$	0.64 (0.39-0.89)	0.05
MR-pro-ADM$_{t72}$	0.76 (0.50-1.0)	0.005
MR-pro-ADM$_{\%change}$	0.74 (0.58-0.91)	0.002
CRP$_{t0}$	0.38 (0.11-0.65)	0.96
PCT$_{t0}$	0.52 (0.28-0.77)	0.23
Lactate$_{t0}$	0.63 (0.38-0.88)	0.008

Supplementary Table S3. ROC curve analysis in patients with a pulmonary infective focus. P-values are calculated with respect to SOFA score.

	**28-day Mortality**	
	AUC (95%CI)	P-value
SOFA	0.60 (0.38-0.82)	-
APACHEII	0.76 (0.55-0.97)	0.01
MR-pro-ADM$_{t0}$	0.56 (0.32-0.79)	0.77
MR-pro-ADM$_{t72}$	0.70 (0.44-0.96)	0.47
MR-pro-ADM$_{\%change}$	0.78 (0.62-0.93)	0.19
CRP$_{t0}$	0.61 (0.38-0.83)	0.92
PCT$_{t0}$	0.65 (0.42-0.87)	0.65
Lactate$_{t0}$	0.67 (0.45-0.89)	0.70

Supplementary Table S4. Logistic regression analysis.

Variable	Adjustment	OR (95% CI)	p-value	LR test for added value
**MR-proADM$_{t0}$**	-	1.4 (1.1-1.9)	0.02	-
	age	1.6 (1.1-2.3)	0.01	35.5%
	SOFA score	1.3 (0.9-1.8)	0.13	-
	APACHEII	1.12 (0.79-1.57)	0.53	-
**MR-proADM$_{t0}$ ≥2.78**	-	4.4 (1.6-12.5)	0.005	-
	age	4.4 (1.5-12.8)	0.008	39.4%
	SOFA score	3.8 (1.2-11.5)	0.02	58.1%
	APACHEII	2.1 (0.7-6.6)	0.22	-
**MR-proADM$_{t72}$**	-	2.0 (1.4-2.9)	<0.001	-
	age	2.0 (1.4-3.0)	<0.001	49.0%
	SOFA score	2.2 (1.4-3.4)	<0.001	82.8%
	APACHEII	1.6 (1.2-2.3)	0.005	26.7%
**MR-proADM$_{t72}$≥2.70**	-	6.7 (2.2-20.3)	<0.001	-
	age	7.7 (2.4-25.2)	<0.001	37.8%
	SOFA score	7.3 (2.1-24.9)	0.002	71.0%
	APACHEII	3.0 (0.8-10.6)	0.09	-
**MR-proADM$_{\%change}$**	-	34.8 (2.3-526.3)	<0.001	-
	age	41.4 (2.3-751.3)	<0.001	46.3%
	SOFA score	52.6 (3.2-853.7)	<0.001	84.5%
	APACHEII	55.2 (2.4-1279)	<0.001	38.0%
**MR-proADM$_{\%change}$ ≥-15.2%**	-	7.0 (2.1-23.3)	0.002	-
	age	7.1 (2.1-24.2)	0.002	34.6%
	SOFA score	7.9 (2.3-27.2)	0.001	75.2%
	APACHEII	10.1 (2.5-41.9)	0.001	32.1%

Supplementary Table S5. Logistic regression analysis in patients with septic shock.

Variable	Adjustment	OR (95%CI)	p-value	LR test for added value
**MR-pro-ADM$_{t0}$**	-	1.5 (0.7-3.0)	0.27	-
**MR-pro-ADM$_{t0}$ ≥2.78**	-	5.4 (0.6-49.1)	0.14	-
**MR-proADM$_{t72}$**	-	4.2 (1.2-15.5)	0.03	-
	age	4.6 (1.0-21.6)	0.05	-
	SOFA	5.4 (1.2-24.3)	0.03	78.6%
	APACHEII	5.6 (1.4-22.1)	0.01	19.9%
**MR-proADM$_{t72}$≥2.7**	-	4.0 (0.7-23.4)	0.12	-
**%delta proADM**	-	1.1 (0.7-1.8)	0.74	-
**%delta proADM >-15.21%**	-	6.3 (1.1-37.8)	0.04	-
	age	10.1 (1.2-84.2)	0.03	37.4%
	SOFA	5.3 (0.9-33.4)	0.07	-
	APACHEII	14.5 (1.3-168.6)	0.03	42.3%

Supplementary Table S6. Logistic regression analysis in patients with a pulmonary infective focus.

Variable	Adjustment	OR (95%CI)	p-value	LR test for added value
**MR-pro-ADM$_{t0}$**	-	1.4 (0.4-4.4)	0.86	-
**MR-pro-ADM$_{t0}$ ≥2.78**	-	4.9 (1.1-20.9)	0.03	-
	age	5.9 (1.3-27.4)	0.02	70.4%
	SOFA	4.4 (0.9-21.0)	0.06	-
	APACHEII	1.8 (0.3-10.2)	0.5	-
**MR-proADM$_{t72}$**	-	1.5 (1.0-2.3)	0.04	-
	age	1.6 (1.0-2.4)	0.04	58.4%
	SOFA	1.6 (0.9-2.7)	0.07	-
	APACHEII	1.1 (0.7-1.9)	0.7	-
**MR-proADM$_{t72}$≥2.7**	-	15.0 (2.6-87.6)	0.003	-
	age	24.7 (2.8-217.0)	0.004	81.7%
	SOFA	17.2 (2.4-121.3)	0.004	88.7%
	APACHEII	6.0 (0.76-47.2)	0.09	-
**%delta proADM**	-	37.3 (1.2-1180)	0.008	-
	age	104.9 (1.6-6888)	0.04	81.7%
	SOFA	53.2 (1.5-1936)	0.03	90.5%
	APACHEII	40.3 (0.7-2441)	0.09	-
**%delta proADM >-15.21%**	-	10.3 (1.8-60.3)	0.01	-
	age	15.8 (2.3-107.9)	0.005	37.4%
	SOFA	10.6 (1.8-62.9)	0.009	87.0%
	APACHEII	16.6 (1.6-171.6)	0.02	51.3%

Supplementary Figure S1. Flow diagram of the study.

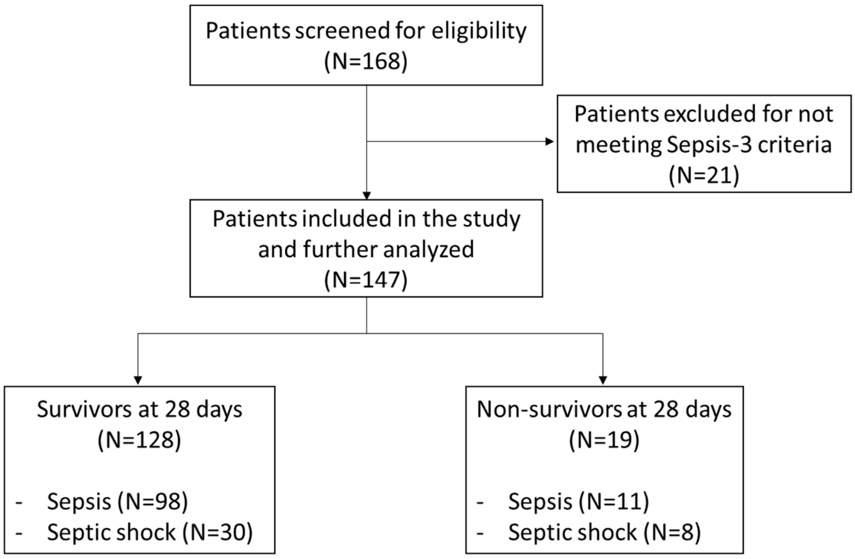

Supplementary Figure S2. Dose-response plot for 28-day mortality predicted by MR-proADM$_{t0}$ (panel A) and MR-proADM$_{t72}$ (panel B) at varying levels of the APACHEII score.

A

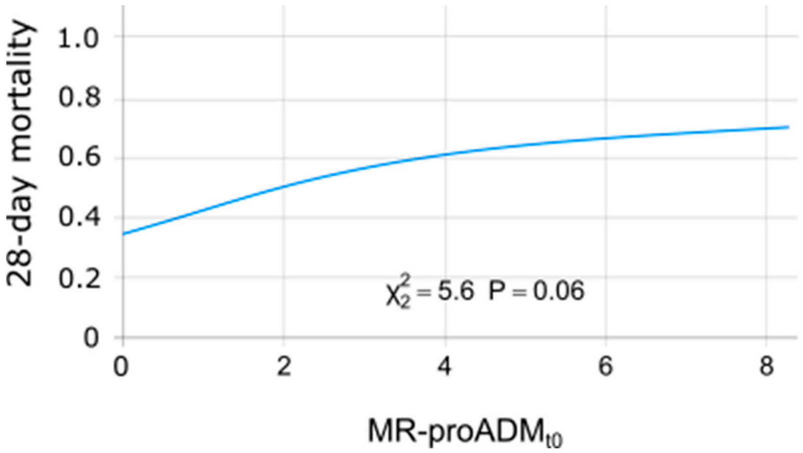

B

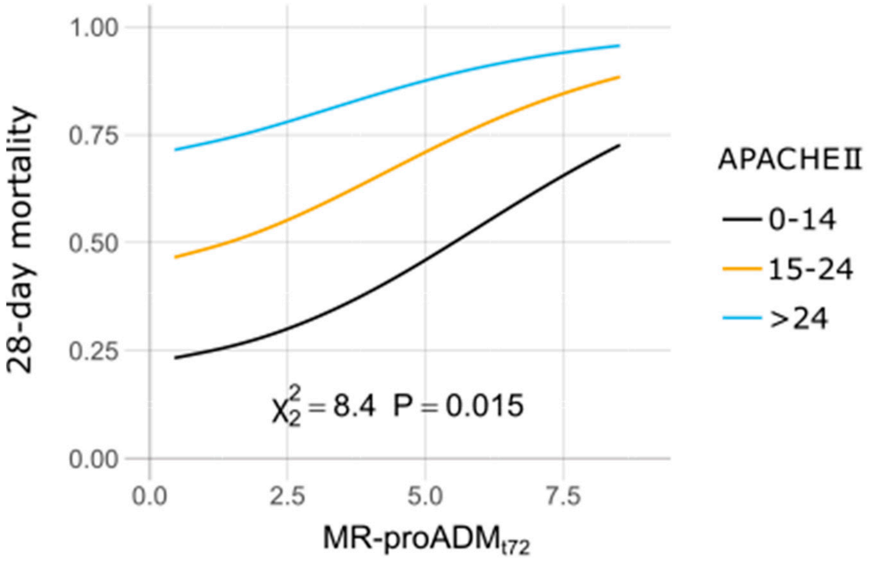

Supplement: Supplementary file 1 [file biomedicines-10-00719-s001.zip › biomedicines-1631418-supplementary.pdf]
